# 5-(3-Methyl­phen­yl)-3-phenyl-1,2-oxazole

**DOI:** 10.1107/S1600536811020198

**Published:** 2011-06-04

**Authors:** B. Balakrishnan, C. Praveen, P. R. Seshadri, P. T. Perumal

**Affiliations:** a Department of Physics, P. T. Lee Chengalvaraya Naicker College of Engineering & Technology, Kancheepuram 631 502, India; b Organic Chemistry Division, Central Leather Research Institute, Chennai 600 020, India; c PostGraduate & Research Department of Physics, Agurchand Manmull Jain College, Chennai 600 114, India

## Abstract

In the title compound, C_16_H_13_NO, the isoxazole ring makes dihedral angles of 16.64 (7)° with 3-methyl­phenzyl ring and 17.60 (7)° with the unsubstituted phenyl ring.

## Related literature

For general background to isoxazole derivatives, see: Sperry & Wright (2005 ▶); Krogsgaard-Larsen *et al.* (1996 ▶); Deng *et al.* (2009 ▶); Talley (1999 ▶).
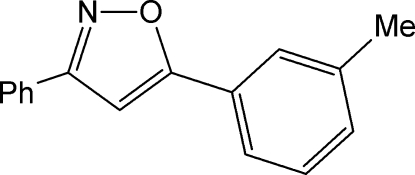


## Experimental

### 

#### Crystal data

C_16_H_13_NO
*M* *_r_* = 235.27
Orthorhombic, 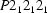

*a* = 5.8052 (2) Å
*b* = 7.7010 (3) Å
*c* = 27.4363 (8) Å
*V* = 1226.56 (7) Å^3^
*Z* = 4
Mo *K*α radiation
μ = 0.08 mm^−1^
*T* = 293 K
0.30 × 0.25 × 0.20 mm

#### Data collection

Bruker Kappa APEXII area-detector diffractometer
Absorption correction: multi-scan (*SADABS*; Bruker, 2004 ▶) *T*_min_ = 0.977, *T*_max_ = 0.984
14583 measured reflections
1599 independent reflections
1302 reflections with *I* > 2σ(*I*)
*R*_int_ = 0.034

#### Refinement

*R*[*F* ^2^ > 2σ(*F* ^2^)] = 0.039
*wR*(*F* ^2^) = 0.109
*S* = 1.08
1599 reflections
164 parameters
H-atom parameters constrained
Δρ_max_ = 0.12 e Å^−3^
Δρ_min_ = −0.18 e Å^−3^

### 

Data collection: *APEX2* (Bruker, 2004 ▶); cell refinement: *SAINT* (Bruker, 2004 ▶); data reduction: *SAINT*; program(s) used to solve structure: *SHELXS97* (Sheldrick, 2008 ▶); program(s) used to refine structure: *SHELXL97* (Sheldrick, 2008 ▶); molecular graphics: *ORTEP-3* (Farrugia, 1997 ▶) and *PLATON* (Spek, 2009 ▶); software used to prepare material for publication: *SHELXL97* and *PLATON*.

## Supplementary Material

Crystal structure: contains datablock(s) I, global. DOI: 10.1107/S1600536811020198/bt5513sup1.cif

Structure factors: contains datablock(s) I. DOI: 10.1107/S1600536811020198/bt5513Isup2.hkl

Supplementary material file. DOI: 10.1107/S1600536811020198/bt5513Isup3.cml

Additional supplementary materials:  crystallographic information; 3D view; checkCIF report

## Figures and Tables

**Fig. 1.**
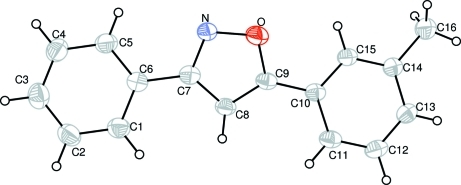
Molecular structure of the title compound, showing 30% probability displacement ellipsoids.

## supplementary crystallographic information

### Comment

Isoxazoles are an important class of heteroaromatic molecules which are components in a variety of natural products and medicinally useful compounds (Sperry *et al.*, 2005). For example, the natural product ibotenic acid, an active constituent of the psychotrophic fly agaric mushroom amanita muscaria, acts at both ionotropic and metabotropic glutamate receptor subtypes (Krogsgaard-Larsen *et al.*, 1996). Isoxazole systems have also been targeted in synthetic investigations for their known bological and pharmacological properties such as hypoglycemic, anti-inflammatory and anti-bacterial activities. The growing interest in such analogues also rises from their high potential value as antiviral agents (Deng *et al.*, 2009). Valdecoxib is a nonsteroidal anti-inflammatory drug used in the treatment of osteoarthritis, rheumatoid arthritis and powerful menstration and menstrual symptoms (Talley, 1999). In the title compound  the isoxazole ring makes a dihedral angle of 16.64 (7)° with methyl benzyl ring (C10/C11/C12/C13/C14/C15/C16) and a dihedral angle of 17.60 (7)° with the phenyl ring (C1/C2/C3/C4/C5/C6) attached to the planar isoxazole moiety.

### Experimental

To a solution of 1-phenyl-3-*m*-tolyl-propynone oxime (235 mg, 1.0(mmol) in dry dichloromethane (1 ml) wasadded AuCl3 (3.03 mg, 1mol%) under N2 atmosphere and stirred for 10 min. After completion of the reaction as indicated by TLC the reaction mixture was concertrated under reduced pressure and purified by column chromatography over silica gel(100–200mech) Et 0 Ac/hexane to afford the pure product.

### Refinement

Due to the absence of anomalous scatterers, Friedel pairs were merged. All H atoms were positioned geometrically and allowed to ride on their parent atoms, with C—H = 0.93–0.97 Å and U_iso_(H) = 1.5*U*_eq_(C) for methyl H atoms and 1.2*U*_eq_(C) for other H atoms.

### Crystal data

**Table:**

C_16_H_13_NO	*D*_x_ = 1.274 Mg m^−^^3^
*M**_r_* = 235.27	Mo *K*α radiation, λ = 0.71073 Å
Orthorhombic, *P*2_1_2_1_2_1_	Cell parameters from 14583 reflections
*a* = 5.8052 (2) Å	θ = 1.5–27.1°
*b* = 7.7010 (3) Å	µ = 0.08 mm^−^^1^
*c* = 27.4363 (8) Å	*T* = 293 K
*V* = 1226.56 (7) Å^3^	Block, colourless
*Z* = 4	0.30 × 0.25 × 0.20 mm
*F*(000) = 496	

### Data collection

**Table:**

Bruker Kappa APEXII area-detector diffractometer	1599 independent reflections
Radiation source: fine-focus sealed tube	1302 reflections with *I* > 2σ(*I*)
graphite	*R*_int_ = 0.034
ω scans	θ_max_ = 27.1°, θ_min_ = 1.5°
Absorption correction: multi-scan (*SADABS*; Bruker, 2004)	*h* = −7→6
*T*_min_ = 0.977, *T*_max_ = 0.984	*k* = −8→9
14583 measured reflections	*l* = −35→35

### Refinement

**Table:**

Refinement on *F*^2^	Primary atom site location: structure-invariant direct methods
Least-squares matrix: full	Secondary atom site location: difference Fourier map
*R*[*F*^2^ > 2σ(*F*^2^)] = 0.039	Hydrogen site location: inferred from neighbouring sites
*wR*(*F*^2^) = 0.109	H-atom parameters constrained
*S* = 1.08	*w* = 1/[σ^2^(*F*_o_^2^) + (0.0547*P*)^2^ + 0.1446*P*] where *P* = (*F*_o_^2^ + 2*F*_c_^2^)/3
1599 reflections	(Δ/σ)_max_ = 0.003
164 parameters	Δρ_max_ = 0.12 e Å^−^^3^
0 restraints	Δρ_min_ = −0.18 e Å^−^^3^

### Special details

**Table:**

Geometry. All e.s.d.'s (except the e.s.d. in the dihedral angle between two l.s. planes) are estimated using the full covariance matrix. The cell e.s.d.'s are taken into account individually in the estimation of e.s.d.'s in distances, angles and torsion angles; correlations between e.s.d.'s in cell parameters are only used when they are defined by crystal symmetry. An approximate (isotropic) treatment of cell e.s.d.'s is used for estimating e.s.d.'s involving l.s. planes.
Refinement. Refinement of *F*^2^ against ALL reflections. The weighted *R*-factor *wR* and goodness of fit *S* are based on *F*^2^, conventional *R*-factors *R* are based on *F*, with *F* set to zero for negative *F*^2^. The threshold expression of *F*^2^ > σ(*F*^2^) is used only for calculating *R*-factors(gt) *etc*. and is not relevant to the choice of reflections for refinement. *R*-factors based on *F*^2^ are statistically about twice as large as those based on *F*, and *R*- factors based on ALL data will be even larger.

### Fractional atomic coordinates and isotropic or equivalent isotropic displacement parameters (Å^2^)

**Table:**

	*x*	*y*	*z*	*U*_iso_*/*U*_eq_	
N	0.3214 (3)	0.4492 (3)	0.09265 (7)	0.0550 (5)	
O	0.3690 (3)	0.3988 (3)	0.14108 (6)	0.0662 (5)	
C9	0.5947 (4)	0.3630 (3)	0.14404 (8)	0.0492 (6)	
C10	0.6819 (4)	0.3105 (3)	0.19162 (8)	0.0471 (5)	
C14	0.6336 (4)	0.2897 (3)	0.27920 (8)	0.0498 (5)	
C7	0.5182 (4)	0.4406 (3)	0.06915 (8)	0.0505 (5)	
C15	0.5545 (4)	0.3395 (3)	0.23393 (8)	0.0491 (5)	
H15	0.4121	0.3941	0.2315	0.059*	
C13	0.8460 (4)	0.2078 (3)	0.28208 (9)	0.0553 (6)	
H13	0.9016	0.1721	0.3123	0.066*	
C8	0.6941 (4)	0.3875 (3)	0.09969 (8)	0.0526 (6)	
H8	0.8483	0.3718	0.0916	0.063*	
C1	0.7032 (5)	0.4368 (3)	−0.01203 (9)	0.0650 (7)	
H1	0.8257	0.3757	0.0015	0.078*	
C12	0.9760 (5)	0.1786 (3)	0.24080 (10)	0.0603 (6)	
H12	1.1181	0.1237	0.2434	0.072*	
C11	0.8968 (4)	0.2303 (3)	0.19575 (10)	0.0556 (6)	
H11	0.9863	0.2119	0.1681	0.067*	
C6	0.5200 (4)	0.4874 (3)	0.01707 (8)	0.0506 (5)	
C5	0.3401 (5)	0.5778 (3)	−0.00369 (9)	0.0627 (7)	
H5	0.2162	0.6123	0.0155	0.075*	
C16	0.4918 (5)	0.3241 (4)	0.32435 (9)	0.0708 (8)	
H16A	0.4844	0.4469	0.3302	0.106*	
H16B	0.5620	0.2679	0.3518	0.106*	
H16C	0.3390	0.2795	0.3198	0.106*	
C4	0.3426 (5)	0.6172 (4)	−0.05246 (10)	0.0718 (8)	
H4	0.2208	0.6784	−0.0662	0.086*	
C3	0.5239 (5)	0.5667 (4)	−0.08088 (10)	0.0739 (8)	
H3	0.5248	0.5934	−0.1139	0.089*	
C2	0.7041 (6)	0.4771 (4)	−0.06096 (10)	0.0728 (8)	
H2	0.8272	0.4432	−0.0804	0.087*	

### Atomic displacement parameters (Å^2^)

**Table:**

	*U*^11^	*U*^22^	*U*^33^	*U*^12^	*U*^13^	*U*^23^
N	0.0399 (10)	0.0734 (13)	0.0517 (11)	0.0032 (11)	0.0004 (9)	0.0030 (10)
O	0.0460 (9)	0.0866 (13)	0.0659 (11)	0.0012 (10)	0.0061 (9)	0.0003 (10)
C9	0.0397 (11)	0.0458 (12)	0.0622 (14)	−0.0024 (10)	0.0069 (10)	−0.0042 (11)
C10	0.0386 (10)	0.0410 (10)	0.0618 (13)	−0.0057 (10)	0.0035 (11)	−0.0007 (10)
C14	0.0464 (12)	0.0423 (11)	0.0607 (13)	−0.0042 (10)	0.0005 (11)	0.0011 (10)
C7	0.0461 (12)	0.0453 (11)	0.0599 (13)	−0.0042 (11)	0.0024 (11)	−0.0036 (10)
C15	0.0414 (12)	0.0426 (11)	0.0634 (14)	0.0008 (10)	0.0032 (10)	0.0021 (10)
C13	0.0481 (12)	0.0482 (12)	0.0696 (15)	−0.0022 (12)	−0.0060 (12)	0.0048 (11)
C8	0.0405 (11)	0.0570 (14)	0.0604 (14)	0.0020 (11)	0.0069 (11)	0.0016 (11)
C1	0.0630 (16)	0.0617 (15)	0.0702 (17)	0.0093 (14)	0.0084 (14)	0.0007 (13)
C12	0.0431 (12)	0.0516 (13)	0.0862 (18)	0.0046 (11)	−0.0021 (13)	0.0033 (13)
C11	0.0423 (11)	0.0507 (13)	0.0739 (16)	−0.0010 (11)	0.0092 (11)	−0.0019 (12)
C6	0.0500 (13)	0.0459 (11)	0.0558 (13)	−0.0033 (11)	0.0016 (11)	−0.0053 (10)
C5	0.0538 (14)	0.0686 (16)	0.0658 (16)	0.0088 (14)	−0.0002 (13)	−0.0047 (12)
C16	0.0697 (17)	0.0822 (19)	0.0605 (14)	0.0098 (17)	−0.0012 (14)	−0.0001 (14)
C4	0.0721 (18)	0.0784 (18)	0.0648 (16)	0.0102 (17)	−0.0104 (15)	−0.0002 (14)
C3	0.095 (2)	0.0701 (17)	0.0569 (15)	0.0028 (18)	−0.0021 (16)	−0.0022 (13)
C2	0.0784 (19)	0.0758 (18)	0.0644 (16)	0.0096 (17)	0.0163 (15)	−0.0032 (14)

### Geometric parameters (Å, °)

**Table:**

N—C7	1.314 (3)	C1—C6	1.386 (3)
N—O	1.412 (2)	C1—H1	0.9300
O—C9	1.341 (3)	C12—C11	1.378 (3)
C9—C8	1.360 (3)	C12—H12	0.9300
C9—C10	1.457 (3)	C11—H11	0.9300
C10—C15	1.394 (3)	C6—C5	1.378 (3)
C10—C11	1.397 (3)	C5—C4	1.372 (4)
C14—C15	1.379 (3)	C5—H5	0.9300
C14—C13	1.387 (3)	C16—H16A	0.9600
C14—C16	1.511 (3)	C16—H16B	0.9600
C7—C8	1.383 (3)	C16—H16C	0.9600
C7—C6	1.474 (3)	C4—C3	1.366 (4)
C15—H15	0.9300	C4—H4	0.9300
C13—C12	1.379 (4)	C3—C2	1.367 (4)
C13—H13	0.9300	C3—H3	0.9300
C8—H8	0.9300	C2—H2	0.9300
C1—C2	1.378 (4)		
			
C7—N—O	106.11 (18)	C11—C12—H12	119.8
C9—O—N	107.76 (18)	C13—C12—H12	119.8
O—C9—C8	109.4 (2)	C12—C11—C10	119.9 (2)
O—C9—C10	116.8 (2)	C12—C11—H11	120.0
C8—C9—C10	133.8 (2)	C10—C11—H11	120.0
C15—C10—C11	118.5 (2)	C5—C6—C1	119.0 (2)
C15—C10—C9	121.2 (2)	C5—C6—C7	121.3 (2)
C11—C10—C9	120.4 (2)	C1—C6—C7	119.7 (2)
C15—C14—C13	118.3 (2)	C4—C5—C6	120.4 (2)
C15—C14—C16	120.6 (2)	C4—C5—H5	119.8
C13—C14—C16	121.2 (2)	C6—C5—H5	119.8
N—C7—C8	111.1 (2)	C14—C16—H16A	109.5
N—C7—C6	118.0 (2)	C14—C16—H16B	109.5
C8—C7—C6	130.9 (2)	H16A—C16—H16B	109.5
C14—C15—C10	121.9 (2)	C14—C16—H16C	109.5
C14—C15—H15	119.0	H16A—C16—H16C	109.5
C10—C15—H15	119.0	H16B—C16—H16C	109.5
C12—C13—C14	120.9 (2)	C3—C4—C5	120.1 (3)
C12—C13—H13	119.5	C3—C4—H4	119.9
C14—C13—H13	119.5	C5—C4—H4	119.9
C9—C8—C7	105.7 (2)	C4—C3—C2	120.3 (3)
C9—C8—H8	127.2	C4—C3—H3	119.8
C7—C8—H8	127.2	C2—C3—H3	119.8
C2—C1—C6	120.1 (3)	C3—C2—C1	120.0 (3)
C2—C1—H1	120.0	C3—C2—H2	120.0
C6—C1—H1	120.0	C1—C2—H2	120.0
C11—C12—C13	120.5 (2)		
			
C7—N—O—C9	0.3 (3)	C6—C7—C8—C9	179.6 (2)
N—O—C9—C8	−0.2 (3)	C14—C13—C12—C11	−0.1 (4)
N—O—C9—C10	179.04 (18)	C13—C12—C11—C10	−1.0 (4)
O—C9—C10—C15	−16.3 (3)	C15—C10—C11—C12	1.4 (3)
C8—C9—C10—C15	162.8 (3)	C9—C10—C11—C12	−178.9 (2)
O—C9—C10—C11	164.0 (2)	C2—C1—C6—C5	0.1 (4)
C8—C9—C10—C11	−16.9 (4)	C2—C1—C6—C7	178.7 (3)
O—N—C7—C8	−0.2 (3)	N—C7—C6—C5	16.0 (3)
O—N—C7—C6	−179.81 (18)	C8—C7—C6—C5	−163.5 (3)
C13—C14—C15—C10	−0.3 (3)	N—C7—C6—C1	−162.6 (2)
C16—C14—C15—C10	179.6 (2)	C8—C7—C6—C1	17.9 (4)
C11—C10—C15—C14	−0.8 (3)	C1—C6—C5—C4	−0.1 (4)
C9—C10—C15—C14	179.6 (2)	C7—C6—C5—C4	−178.7 (2)
C15—C14—C13—C12	0.7 (3)	C6—C5—C4—C3	0.1 (4)
C16—C14—C13—C12	−179.2 (2)	C5—C4—C3—C2	−0.2 (4)
O—C9—C8—C7	0.1 (3)	C4—C3—C2—C1	0.2 (4)
C10—C9—C8—C7	−179.0 (2)	C6—C1—C2—C3	−0.1 (4)
N—C7—C8—C9	0.1 (3)		

### Hydrogen-bond geometry (Å, °)

**Table:**

*D*—H···*A*	*D*—H	H···*A*	*D*···*A*	*D*—H···*A*
C15—H15···O	0.93	2.49	2.803 (3)	100.
